# Supplementary material for: Epigenetic dysregulation of IRF9 drives excessive interferon signaling in COPD
Source: EMBO Mol Med. 2026 Feb 23;18(4):1202–34. doi: 10.1038/s44321-026-00386-9 (PMC13083945; doi:10.1038/s44321-026-00386-9)
Supplement: Supplementary file 14 — Expanded View Figures [file 44321_2026_386_MOESM14_ESM.pdf]

Expanded View Figures

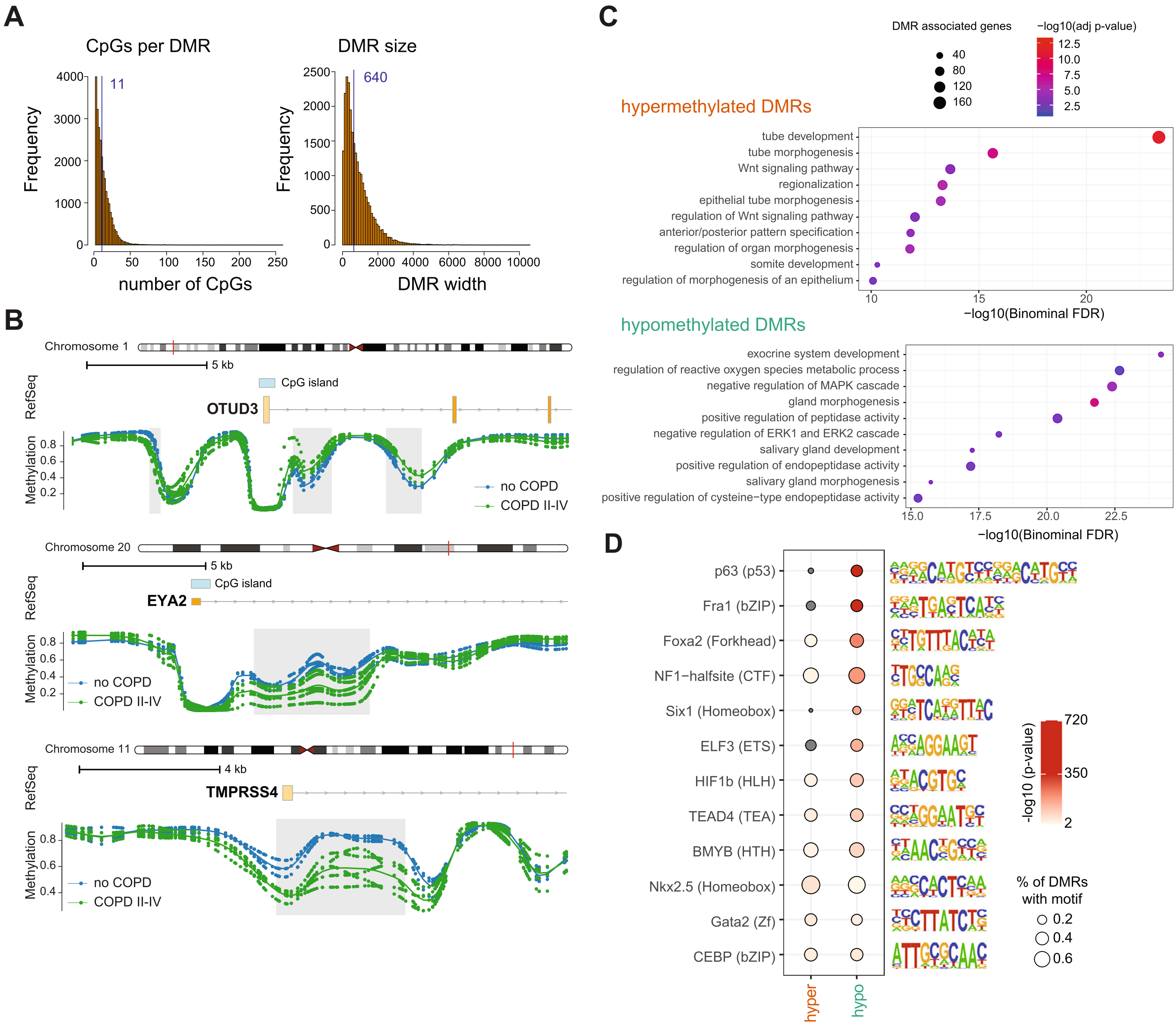

Figure EV1. Genome-wide DNA methylation changes occur at regulatory regions in AT2 cells during COPD (supporting information for Fig. 2).

(**A**) Number of CpG sites (left panel) and the width of DMRs (right panel) identified between no COPD and COPD II–IV. Median values are indicated in the histograms in dark blue. (**B**) Detailed view of DMRs showing the methylation profiles of no COPD ($n = 3$) and COPD II–IV ($n = 5$) samples at the indicated genomic regions. DMR locations are highlighted as gray boxes. (**C**) Functional annotation of genes located next to hypermethylated (top) and hypomethylated (bottom) DMRs using GREAT. Hits were sorted according to the binomial adjusted *P* value, and the top 10 hits are shown. The adjusted *P* value is indicated by the color code, and the number of DMR-associated genes is indicated by the node size. (**D**) Transcription factor motif enrichment in hypermethylated (left) and hypomethylated (right) DMRs. The top motif of each transcription factor family (in brackets) is shown. The node size indicates the percentage of DMRs containing the respective motif, and the color represents the *P* value of the enrichment analysis. Data information: In (**C**), *P* values were calculated using GREAT, which uses a binomial test. The Benjamini–Hochberg method was applied to correct for multiple testing, revealing adjusted *P* values. Exact *P* values are included in Dataset EV4. In (**D**), motif enrichment *P* values were calculated using HOMER, which uses ZOOPS scoring (zero or one occurrence per sequence) coupled with the hypergeometric enrichment calculations. Exact *P* values are included in Dataset EV5.

© The Author(s)

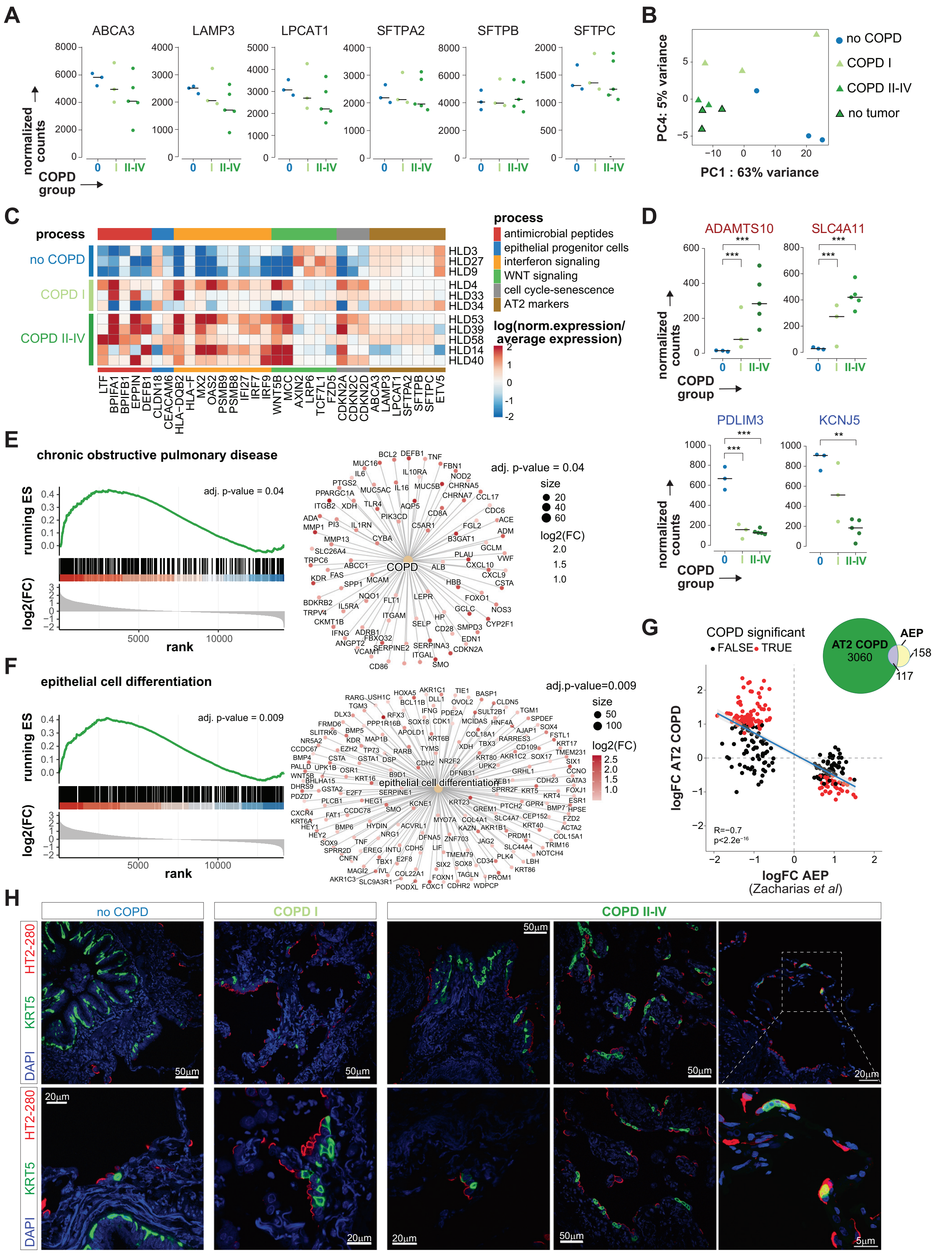
A
ABCA3
LAMP3
LPCAT1
SFTPA2
SFTPB
SFTPC
normalized counts
COPD group
0
I
II-IV
B
no COPD
COPD I
COPD II-IV
no tumor
PC4: 5% variance
PC1 : 63% variance
C
process
no COPD
COPD I
COPD II-IV
HLD3
HLD27
HLD9
HLD4
HLD33
HLD34
HLD53
HLD39
HLD58
HLD14
HLD40
LTF BPIFA1 BPIFB1 EPPIN DEFB1 CLDN18 CEACAM6 HLA-DQB2 HLA-F MX2 OAS2 PSMB9 PSMB8 IFI27 IRF7 IRF9 WNT5B MCC AXIN2 LRP6 TCF7L1 FZD5 CDKN2A CDKN2C CDKN2D ABCA3 LAMP3 LPCAT1 SFTPA2 SFTPB SFTPC ETV5
antimicrobial peptides
epithelial progenitor cells
interferon signaling
WNT signaling
cell cycle-senescence
AT2 markers
log(norm.expression/ average expression)
D
ADAMTS10
SLC4A11
PDLIM3
KCNJ5
normalized counts
COPD group
E
chronic obstructive pulmonary disease
adj. p-value = 0.04
running ES
log2(FC)
rank
COPD
size
log2(FC)
F
epithelial cell differentiation
adj. p-value = 0.009
adj.p-value=0.009
running ES
log2(FC)
rank
epithelial cell differentiation
size
log2(FC)
G
COPD significant
FALSE TRUE
AT2 COPD
3060
AEP
158
117
logFC AT2 COPD
logFC AEP (Zacharias et al)
R=−0.7
p<2.2e−16
H
no COPD
COPD I
COPD II-IV
HT2-280
KRT5
DAPI
50μm
20μm
5μm

Figure EV2. AT2 transcriptome is altered in COPD as disease progresses (Supporting information for Fig. 3).

(**A**) Normalized read counts from RNA-seq data for AT2-specific genes in sorted AT2 cells from each donor (dots). Data points represent normalized counts from no COPD (blue, $n = 3$), COPD I (light green, $n = 3$), and COPD II–IV (dark green, $n = 5$). The group median is shown as a black bar. (**B**) Principal component analysis (PCA) of the 500 most variable genes in RNA-seq. Percentages indicate the proportion of variance explained by PC1 and PC4. COPD I and COPD II–IV samples are represented in light and dark green triangles, respectively, and no COPD samples as blue circles. COPD samples without a cancer background are displayed with a black contour. (**C**) Heatmap showing expression changes of selected genes across all samples. Genes associated with selected processes are shown. The expression deviation from the average expression across all samples (log(norm. expression/average expression)) is indicated by the color code. (**D**) Top upregulated (top, red label) and top downregulated (bottom, blue label) genes in AT2 cells from COPD patients. Normalized read counts from RNA-seq data for the specified genes in each donor (dots). Data points represent normalized counts from no COPD (blue, $n = 3$), COPD I (light green, $n = 3$), and COPD II–IV (dark green, $n = 5$). The group median is shown as a black bar. Adjusted P values were calculated using DESeq2, which uses a negative binomial GLM (generalized linear model) and Wald statistics. Significance: **: adj. P value < 0.01, ***: adj. P value < 0.001. Specific adj. P values of either COPD II–IV or COPD I compared to no COPD are as follows for each gene: ADAMTS10: <0.001, <0.001; SLC4A11: <0.001, <0.001; PDLIM3: <0.001, <0.001; KCNJ5: 0.001, 0.72. (**E**, **F**) Gene set enrichment analysis (GSEA) results of the gene expression of AT2 cells from COPD II–IV vs no COPD donors. ES enrichment score, NES normalized enrichment score, FDR false discovery rate. GSEA of genes associated with (**E**) chronic obstructive pulmonary diseases (Diseases Ontology ID: 3083) or (**F**) epithelial cell differentiation (Gene Ontology: GO:0030855). Genes were sorted based on the log2(fold change) in COPD II–IV (bottom panel). The P value of the GSEA is indicated in the top right corner and calculated empirically from a null distribution of enrichment scores generated by permutations. Gene network plot of the respective terms shows genes associated with the term and driving the enrichment score (leading edge). The beige nodes symbolize enriched terms. The lines connecting the nodes denote the specific genes associated with each term. The color of the gene nodes signifies the expression change in severe COPD compared to no COPD. (**G**) Top, scatter plot showing genes differentially expressed in alveolar epithelial progenitor (AEP) cells from (Zacharias et al, 2018) compared to AT2 (Zacharias AEP) and their correlation to AT2 cells in COPD II–IV compared to AT2 from no COPD donors from our study (AT2 COPD). DEG in AEP were determined with the same pipeline and cutoffs (red dots; FDR of 10% and | log2(fold change) | >0.5) as used for RNA-seq of no COPD vs COPD II–IV (see "Methods"). DEGs in COPD II–IV are highlighted as red dots. The blue diagonal represents the linear regression between log2(fold-changes) in COPD II–IV against no COPD and AEP against AT2. Shaded areas are confidence intervals of the correlation coefficient at 95%. P value < $2.2e^{-16}$ was derived from linear regression analysis and the Pearson correlation coefficient ($R = -0.7$) is indicated. Right corner, Venn diagram indicating the overlap of DEG in AT2 COPD and AEP cells from (Zacharias et al, 2018) compared to AT2 cells. (**H**) Representative immunofluorescence staining images of HT2-280 and KRT5 expression in FFPE human lung tissue slices from no COPD, COPD I and COPD II–IV donors. The zoomed-in panel (right corner, bottom) demonstrates the presence of rare HT2-280/KRT5 double-positive cells in the alveoli of COPD patients. Slides were counterstained with DAPI, scale bars = 50 µm, 20 µm or 5 µm, as displayed in images.

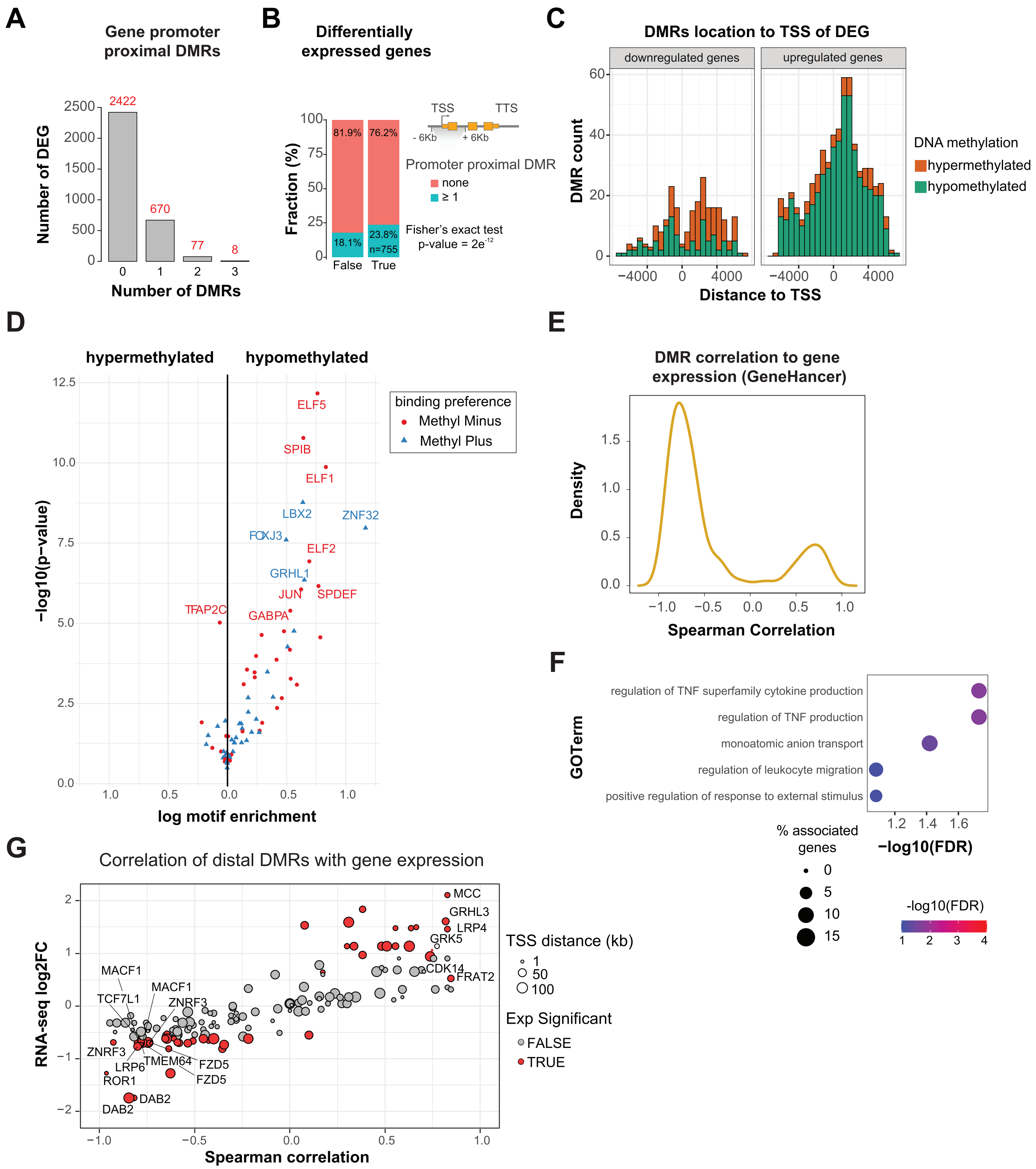
A
Gene promoter proximal DMRs
Number of DEG
Number of DMRs
2422
670
77
8
B
Differentially expressed genes
Fraction (%)
81.9%
76.2%
18.1%
23.8%
n=755
False
True
TSS
TTS
- 6Kb
+ 6Kb
Promoter proximal DMR
none
≥ 1
Fisher's exact test
p-value = 2e-12
C
DMRs location to TSS of DEG
downregulated genes
upregulated genes
DMR count
Distance to TSS
DNA methylation
hypermethylated
hypomethylated
D
hypermethylated
hypomethylated
−log10(p−value)
log motif enrichment
binding preference
Methyl Minus
Methyl Plus
ELF5
SPIB
ELF1
LBX2
ZNF32
FOXJ3
ELF2
GRHL1
JUN
SPDEF
TFAP2C
GABPA
E
DMR correlation to gene expression (GeneHancer)
Density
Spearman Correlation
F
GOTerm
regulation of TNF superfamily cytokine production
regulation of TNF production
monoatomic anion transport
regulation of leukocyte migration
positive regulation of response to external stimulus
% associated genes
−log10(FDR)
-log10(FDR)
G
Correlation of distal DMRs with gene expression
RNA-seq log2FC
Spearman correlation
MCC
GRHL3
LRP4
GRK5
CDK14
FRAT2
MACF1
TCF7L1
MACF1
ZNRF3
ZNRF3
LRP6
TMEM64
FZD5
FZD5
ROR1
DAB2
DAB2
TSS distance (kb)
Exp Significant
FALSE
TRUE

 © The Author(s)

Figure EV3. Integrated analysis reveals epigenetic regulation of key pathways in COPD.

(Supporting information for Fig. 4). (**A**) DMRs within ± 6 kb from the TSS of DEG were assigned to their corresponding gene. (**B**) Fraction of genes associated with at least one DMR in the promoter proximity (+/− 6 kb from TSS, blue) of non-DEG (left, 18.1%) or DEG (right, 23.8%). DMRs are significantly enriched at DEGs (Fisher's exact test *P* value = $2e^{-12}$). (**C**) Stacked histogram showing location of hyper- and hypomethylated DMRs relative to the TSS of DEGs in downregulated (left) and upregulated (right) genes. (**D**) Enrichment of methylation-sensitive binding motifs at hypo- (right) and hypermethylated (left) DMRs, using DMRs with a high correlation (| Spearman correlation coefficient | >0.5) between methylation and gene expression. Methylation-sensitive motifs were derived from Yin et al (Yin et al, 2017b). Transcription factors, whose binding affinity is impaired upon methylation of their DNA binding motif, are shown in red (binding preference: Methyl Minus), and transcription factors, whose binding affinity upon CpG methylation is increased, are shown in blue (binding preference: Methyl Plus). (**E**) Spearman correlation between gene expression and DMR methylation of DMRs assigned to gene regulatory elements using the GeneHancer database. (**F**) GO-Term overrepresentation analysis of DEGs negatively correlated to DMRs in gene regulatory elements. The adjusted *P* value is indicated by the color code and the percentage number of associated DEGs is indicated by the node size. Exact *P* values are included in Dataset EV10. (**G**) Scatter plot showing distal DMR-DEG pairs associated with Wnt-signaling. Pairs were extracted from GREAT analysis (hypermethylated, DMR-DEG distance <100 kb; see Fig. EV1C). The *Y* axis represents the log2 fold change expression in COPD II–IV, and the X-axis denotes the Spearman correlation of the DEG-DMR pair. Node size indicates the distance of the DMR to the TSS. DEGs are highlighted in red.

© The Author(s)

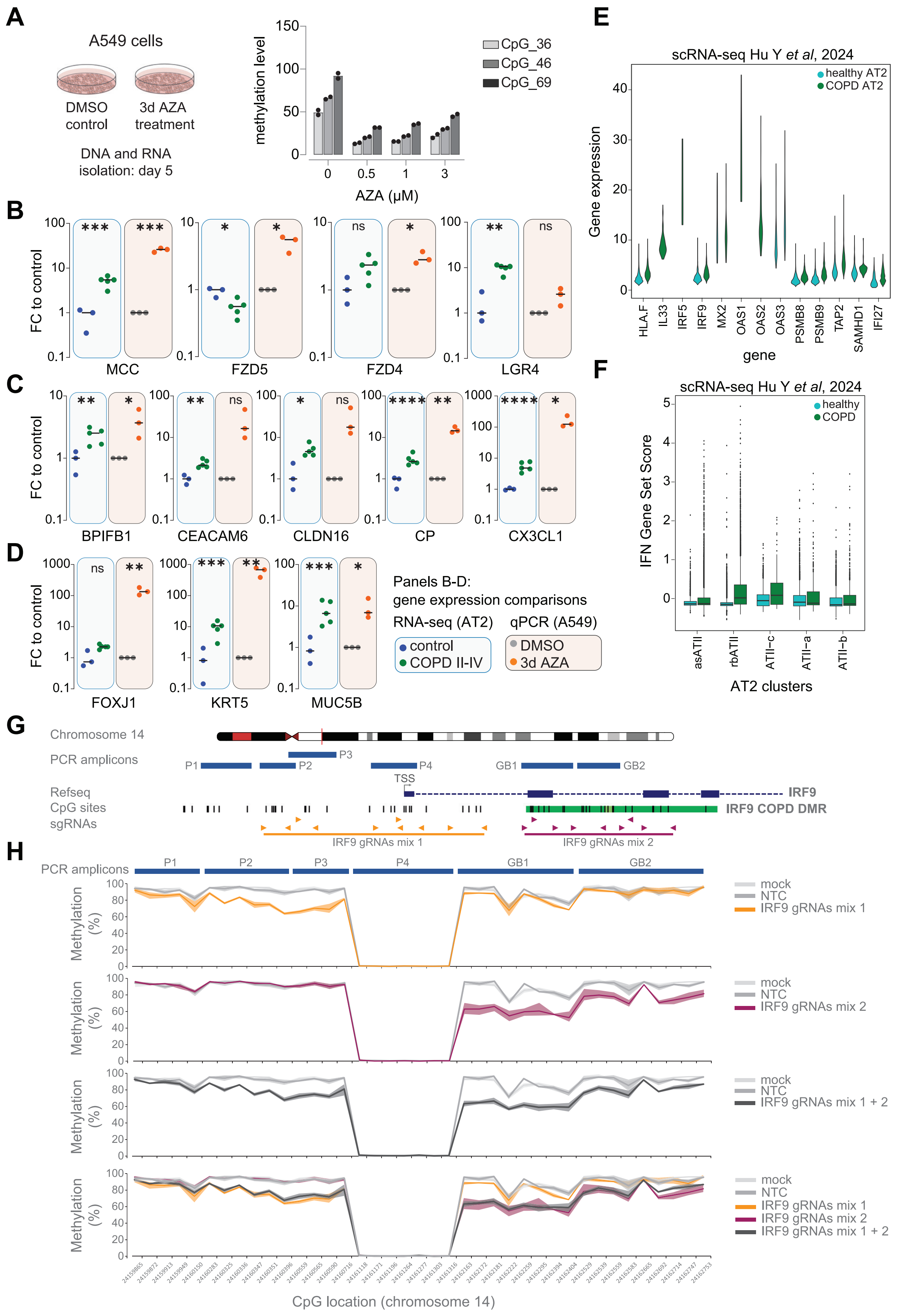
A
A549 cells
DMSO control
3d AZA treatment
DNA and RNA isolation: day 5
methylation level
AZA (μM)
CpG_36
CpG_46
CpG_69
B
FC to control
MCC
FZD5
FZD4
LGR4
C
BPIFB1
CEACAM6
CLDN16
CP
CX3CL1
D
FOXJ1
KRT5
MUC5B
Panels B-D:
gene expression comparisons
RNA-seq (AT2)
qPCR (A549)
control
COPD II-IV
DMSO
3d AZA
E
scRNA-seq Hu Y et al, 2024
healthy AT2
COPD AT2
Gene expression
gene
HLA.F
IL33
IRF5
IRF9
MX2
OAS1
OAS2
OAS3
PSMB8
PSMB9
TAP2
SAMHD1
IFI27
F
scRNA-seq Hu Y et al, 2024
healthy
COPD
IFN Gene Set Score
asATII
rbATII
ATII−c
ATII−a
ATII−b
AT2 clusters
G
Chromosome 14
PCR amplicons
P1
P2
P3
P4
GB1
GB2
TSS
Refseq
CpG sites
sgRNAs
IRF9
IRF9 COPD DMR
IRF9 gRNAs mix 1
IRF9 gRNAs mix 2
H
PCR amplicons
Methylation (%)
mock
NTC
IRF9 gRNAs mix 1
IRF9 gRNAs mix 2
IRF9 gRNAs mix 1 + 2
CpG location (chromosome 14)

 © The Author(s)

Figure EV4. Epigenetic regulation of gene expression in AT2 and A549 cells (Supporting information for Fig. 5).

(**A**) LINE methylation levels in A549 cells treated with the indicated amounts of 5-Aza-2′-deoxycytidine (AZA) and measured at three CpG sites by Mass Array ($n = 2$ biological replicates; barplot indicates the mean value; each data point is indicated). (**B–D**) Fold-change in gene expression of selected genes in AT2 cells in COPD (RNA-seq) and A549 cells treated with 0.5 µM AZA (RT-qPCR) compared to the median of control samples. Genes were selected based on the presence of a DMR and deregulation in AT2 cells in COPD. Left, RNA-seq data from AT2 cells (no COPD, blue, $n = 3$; COPD II–IV, green, $n = 5$), right, A549 treated with AZA (orange, $n = 3$) compared to control DMSO-treated cells (gray, $n = 3$). The group median is shown as a black bar. Selected genes from the Wnt/b-catenin pathway (**B**), antimicrobial peptides, alveolar progenitor (**C**) and airway epithelial markers (**D**) are shown. RNA-seq adjusted (adj.) *P* values were calculated using DESeq2 with negative binomial generalized linear model (GLM) and Wald statistics. *:adj. *P* value < 0.05; **: adj. *P* value < 0.01; ***: adj. *P* value < 0.001. Gene expression was measured by RT-qPCR using DMSO treatment as control (gray) and RPLP0 as housekeeping gene. For A549 samples, each point represents the mean of two technical replicates, and bars represent the median of 3 independent experiments ($n = 3$). Statistical analysis was performed by paired *t* test, FDR-corrected using the Benjamini, Krieger, and Yekutieli two-stage set-up method. Significance: **P* value < 0.05; ***P* value < 0.01. (**E**) Expression values for the indicated genes of the IFN pathway in an external scRNA-seq dataset of AT2 cells from COPD patients and healthy controls (Hu et al, 2024). *Y* axis shows log-normalized gene expression levels. Boxplots middle line corresponds to the median; the lower and upper hinges correspond to first and third quartiles, respectively; the upper whisker extends from the hinge to the largest value no further than 1.5× the interquartile range (or the distance between the first and third quartiles) from the hinge and the lower whisker extends from the hinge to the smallest value at most 1.5x the interquartile range of the hinge. Data beyond the end of the whiskers are called 'outlying' points and are plotted individually. (**F**) Combined gene set score of the genes shown in (**E**) in different subsets of AT2 cells from (Hu et al, 2024). The IFN signature genes were identified in our integrative analysis of TWGBS and RNA-seq in sorted AT2 cells. Boxplots are defined as in (**E**). (**G**) Graphical representation of the IRF9 locus. In dark blue is the IRF9 coding region, with introns and exons represented by dashed and solid boxes, respectively. At the bottom, arrows represent individual gRNAs targeting the IRF9 promoter (orange) and gene body (magenta) regions, with overlapping lines representing the groups of gRNAs that comprise mix 1 and mix 2, respectively. Bisulfite-PCR amplicons targeting IRF9 (blue bars) are depicted as blue boxes, with P1-4 targeting the IRF9 promoter region, and GB1-2 targeting the gene body region. Individual CpG sites are depicted by black bars below the IRF9 coding region, with the light and dark green overlapping region displaying the core and extended differentially methylated regions, respectively, identified in the AT2 COPD T-WGBS data. Genomic positions were extracted from human genome assembly 38 (hg38) using the UCSC genome browser. (**H**) CpG-methylation percentage at individual IRF9 CpG sites in epi-edited A549 cells. The percentage of CpG-methylation for mix 1, 2, and 1 + 2 (orange, magenta, and dark gray) transfected samples are plotted separately against the pUC19 mock (mock) and non-targeted (NTC) transfection controls. For each sample, the opaque line plots the mean value, and the faint surrounding area plots the observed range across repeats. The bisulfite PCR targets are displayed across the top, showing which CpG-sites are sequenced by each target, and how these sites relate to the genomic locations of the sequencing targets seen in panel. Data information: In (**B–D**), the specific q-values for each gene from A549 treated samples compared to DMSO control are as follows: MCC, 0.001865; FZD4, 0.010078; FZD5, 0.010078; LGR4, 0.037818; BPIFB1, 0.027136; CX3CL1, 0.002964; CEACAM6, 0.013600; CLDN16, 0.011915; CP, 0.002817; FOXJ1, 0.037818; KRT5, 0.001715; MUC5B, 0.002530. For the ATII RNA-seq analysis, the specific adjusted *P* values for each gene can be found in Dataset EV6.
